# Recognition of Genetic Conditions After Learning With Images Created Using Generative Artificial Intelligence

**DOI:** 10.1001/jamanetworkopen.2024.2609

**Published:** 2024-03-15

**Authors:** Rebekah L. Waikel, Amna A. Othman, Tanviben Patel, Suzanna Ledgister Hanchard, Ping Hu, Cedrik Tekendo-Ngongang, Dat Duong, Benjamin D. Solomon

**Affiliations:** 1 Medical Genetics Branch, National Human Genome Research Institute, Bethesda, Maryland

## Abstract

**Importance:**

The lack of standardized genetics training in pediatrics residencies, along with a shortage of medical geneticists, necessitates innovative educational approaches.

**Objective:**

To compare pediatric resident recognition of Kabuki syndrome (KS) and Noonan syndrome (NS) after 1 of 4 educational interventions, including generative artificial intelligence (AI) methods.

**Design, Setting, and Participants:**

This comparative effectiveness study used generative AI to create images of children with KS and NS. From October 1, 2022, to February 28, 2023, US pediatric residents were provided images through a web-based survey to assess whether these images helped them recognize genetic conditions.

**Interventions:**

Participants categorized 20 images after exposure to 1 of 4 educational interventions (text-only descriptions, real images, and 2 types of images created by generative AI).

**Main Outcomes and Measures:**

Associations between educational interventions with accuracy and self-reported confidence.

**Results:**

Of 2515 contacted pediatric residents, 106 and 102 completed the KS and NS surveys, respectively. For KS, the sensitivity of text description was 48.5% (128 of 264), which was not significantly different from random guessing (odds ratio [OR], 0.94; 95% CI, 0.69-1.29; *P* = .71). Sensitivity was thus compared for real images vs random guessing (60.3% [188 of 312]; OR, 1.52; 95% CI, 1.15-2.00; *P* = .003) and 2 types of generative AI images vs random guessing (57.0% [212 of 372]; OR, 1.32; 95% CI, 1.04-1.69; *P* = .02 and 59.6% [193 of 324]; OR, 1.47; 95% CI, 1.12-1.94; *P* = .006) (denominators differ according to survey responses). The sensitivity of the NS text-only description was 65.3% (196 of 300). Compared with text-only, the sensitivity of the real images was 74.3% (205 of 276; OR, 1.53; 95% CI, 1.08-2.18; *P* = .02), and the sensitivity of the 2 types of images created by generative AI was 68.0% (204 of 300; OR, 1.13; 95% CI, 0.77-1.66; *P* = .54) and 71.0% (247 of 328; OR, 1.30; 95% CI, 0.92-1.83; *P* = .14). For specificity, no intervention was statistically different from text only. After the interventions, the number of participants who reported being unsure about important diagnostic facial features decreased from 56 (52.8%) to 5 (7.6%) for KS (*P* < .001) and 25 (24.5%) to 4 (4.7%) for NS (*P* < .001). There was a significant association between confidence level and sensitivity for real and generated images.

**Conclusions and Relevance:**

In this study, real and generated images helped participants recognize KS and NS; real images appeared most helpful. Generated images were noninferior to real images and could serve an adjunctive role, particularly for rare conditions.

## Key Points

**Question:**

When compared with other education methods, is exposure to images developed using generative artificial intelligence associated with improved recognition of Kabuki and Noonan syndromes among pediatric residents?

**Findings:**

In this comparative effectiveness study, generative methods were used to create images of fake but realistic-appearing individuals with Kabuki and Noonan syndrome. Through online surveys, generated images were found to help residents recognize these syndromes and improved their confidence in this area compared with text-only descriptions, although real images were most helpful.

**Meaning:**

These findings suggest that generative artificial intelligence could supplement genetics education for pediatric residents by helping teach the recognition of rare conditions.

## Teaser

This comparative effectiveness study explored whether exposure to generative artificial intelligence (AI) images was associated with improved recognition of Noonan and Kabuki syndromes among pediatric residents.

## Introduction

Deep learning (DL), a subfield of artificial intelligence (AI), has become a powerful tool in biomedical research, with strong clinical potential.^1,2,3^ Generative AI is a relatively new branch of DL in which new data can be created through training on existing data sets. One type of generative AI that can be used for image generation uses generative adversarial networks (GANs).^4^ Generated images can be used for purposes such as to enhancing small data sets and increasing data set diversity.^5,6^ Another possible use is medical education.^7,8^ For example, fake images can be created and customized to help expose radiologist or pathologist trainees to many radiographic, magnetic resonance, or hematologic images.^9^

These types of technologies can be advantageous in the field of genetic diseases because they can increase exposure to and recognition of rare conditions across populations.^10^ Genetic disorders and congenital anomalies, which can involve severe clinical sequelae, are individually rare, but in aggregate affect up to 5% of live births; per a recent analysis,^15^ a conservative estimate suggests that up to 5.9% of the population is affected by a rare disease, with 71.9% of these disease being genetic and 88.1% having a pediatric onset.^11,12,13,14,15,16^ However, pediatric residents may have sparse exposure to medical genetics training and insufficient educational resources due to a lack of formal genetics training requirements.^17^ This issue, compounded by the shortage of medical geneticists, necessitates creative solutions to optimize early diagnosis and management of genetic conditions.^18^

Despite this lack of standardized genetics training, some programs have implemented innovative educational strategies. These strategies include immersive learning methods, such as personal genome sequencing, cross-specialty training, didactic courses, or alternative electronic-learning methods. Such approaches may be particularly beneficial for programs with limited contact with medical geneticists.^19,20^ In 1 survey, 87% of pediatric residents agreed that an online module could effectively deliver genetics education,^21^ and an email-based approach helped disseminate genomic information.^22^ Additionally, medical students are interested in AI in medicine.^22,23^ In the spirit of these innovations, we conducted a study to investigate the potential use of generative AI in training pediatric residents to identify specific genetic conditions.

## Methods

### Data Collection and Image Selection

Similar to our previous work,^24,25^ we used publicly available images from individual internet searches based on condition names depicting different individuals with Kabuki syndrome (KS) (OMIM 147920 and 300867)^26^ or Noonan syndrome (NS) (OMIM 163950, 605275, 609942, 610733, 611553, 613706, 615355, 616559, 616564, and 619087) (eTable 1 in Supplement 2).^27^ We chose these syndromes because they are relatively common genetic disorders with recognizable facial features that can be important to diagnose early due to their clinically relevant but often occult impact on organ systems.^26,27^ We also felt that NS may be more known and recognizable to pediatric residents than KS and wanted to contrast results. Data on age and gender of the study participants were not collected in the survey. This comparative effectiveness study was approved as exempt by the National Institutes of Health’s institutional review board because the study used educational tests, surveys, interviews, or observations of public behavior. Informed consent was not required by the institutional review board because the study was formally provided exemption status. The study followed the Strengthening the Reporting of Observational Studies in Epidemiology (STROBE) and International Society for Pharmacoeconomics and Outcomes Research (ISPOR) reporting guidelines.

From the available information for each image, we documented age, gender, and ancestry (eTable 1 in Supplement 2). We collected 278 NS images and 239 KS images. Because we focused on pediatric residents, only pediatric (newborn to approximately 18 years of age) images were used for the survey. The survey was administered from October 1, 2022, to February 28, 2023. To ensure image accuracy, we checked that images (both real and GAN based, as described in the following section) used in the surveys were correctly classified by Face2Gene (FDNA).^28^

### GAN Generation

We fine-tuned StyleGAN2-ADA (Nvidia) on our labeled data set to generate fake images (eFigure 1 in Supplement 1).^4^ Originally, StyleGAN2-ADA was trained on unlabeled data sets. Following our previous work,^25^ we included label embeddings to StyleGAN2-ADA. This embedding consists of 3 vectors: disease, age group, and gender. We describe individuals in terms of gender instead of sex, but we caution interpretation due to incomplete data about sex and gender for all individuals. Although we evaluated the GAN application on just KS and NS, we used images of the other conditions during fine-tuning (eTable 1 in Supplement 2). Because people with different conditions can have similar facial features, these additional images allow us to generate more specific images of people with KS and NS. On the basis of current recommendations for population descriptors and due to incomplete information in the training data, we did not create label embeddings related to population characteristics (eg, genetic ancestry or similarity).^29^

For data preprocessing, we resized the images to 256 × 256 resolution and aligned the faces into similar head sizes and orientations. We further removed the background, which helps remove potential artifacts, such as artifactual hair strands. During fine-tuning, we used batch size 64 (ie, sending 64 randomly chosen images computation). We applied sampling weights so that each disease has roughly equal representation in the batch.^30^

After fine-tuning, we used the label embeddings to generate fake images of a person with a particular condition, age, and gender. To create the transformation strips (eMethods in Supplement 1), we manipulated the disease embedding while keeping age and gender embeddings fixed.^24^ For example, to generate an image of a young girl with KS, we provided a random vector and the label embeddings of KS, young child, and female. To make this generated individual look like an unaffected person, we interpolated the embeddings of KS and unaffected, while keeping the corresponding random vector, the age, and gender embedding unchanged.

### Comparison of Educational Interventions

We compared educational interventions via surveys sent using Qualtrics software (October 2021 to February 2022; Qualtrics). Surveys were specific to either KS or NS. We compared 4 different interventions to assess the efficacy of various educational approaches. Survey arms included (1) text-only description of facial features, (2) text description plus 5 images of real individuals with the condition, (3) text description plus 5 GAN images of the condition, and (4) text description plus 5 GAN transformation strips (eFigure 2 in Supplement 1). Each survey included 12 images of people with the condition of interest (KS or NS) plus 8 images of people with other conditions. Participants were not told the numbers of images of each type or (initially) the types of surveys administered. In preliminary testing, medical genetics residents reported that transformation strips helped them recognize genetic conditions (eMethods in Supplement 1). In addition to checking images through an external classifier as described, clinicians in our group manually reviewed images to ensure they were characteristic.

After the educational intervention, participants were asked to classify (using the term *classify* per AI terminology; in this context, *classify* is synonymous with *categorize* or *identify*) 20 images, as well as rate their confidence level for each classification. Participants answered demographic questions and preintervention and postintervention questions about diagnostic facial features and the impact of age, gender, and ancestry on diagnostics. Example surveys can be found online.^30^

To identify participants, we obtained names of programs through the American Board of Pediatrics and used publicly available email lists. Participants were recruited via email and provided with 2 URL links (1 for KS and 1 for NS surveys) with the option of completing 1 or both surveys. A survey was considered complete if a response was given for each image question.

### Statistical Analysis

Logistic regression for clustered data was performed to assess how different education interventions correlated with participant performance.^31^ Both sensitivity and specificity of identifying KS vs the other conditions (and likewise for NS) were determined. The data were partitioned into 2 subsets, 1 in which the questions pertain to the condition of interest (eg, where the correct answer is “Kabuki syndrome”) and 1 in which the questions pertain to the other conditions (eg, where the correct answer choice is “other syndrome”). We fit logistic regression on these 2 subsets separately. The intercept was set as the text-only intervention. The other regression coefficients described how real-image, GAN, and transformation strip interventions affect the marginal (or population-averaged) performance with respect to the text-only intervention. Because surveys were sent randomly, we assume that the participants were roughly randomized and did not add other coefficients to control for potential confounders. All parameters were approximated via generalized estimation equations using the R library geepack, version 1.3.9.^32^ See eMethods in Supplement 1 for further details.

For sensitivity and specificity, we further test for noninferiority between GAN and transformation strip intervention vs the real images. In this case, the logistic regression intercept is the real-image intervention; the other coefficients represent how GAN and transformation intervention change the odds ratio (OR) with respect to the real-image intervention.

A similar logistic regression strategy was used to measure how confidence level was associated with performance conditioned on each intervention separately. That is, using data from only 1 intervention, we fit logistic regression for clustered data between confidence level and performance. The intercept represents the marginal log odd of identifying the correct condition when the average participant has low to no confidence. The slope represents how this log odd changes when the average participant is confident or highly confident. If this slope is statistically insignificant, this intervention would have potentially falsely inflated the self-assurance of the participants. To analyze questions asked only once per participant (eg, important diagnostic facial features and influence of age, ancestry, and gender), we applied a 2-sample *t* test. A 2-tailed *P* < .05 was considered statistically significant.

## Results

### Comparison of Education Interventions

**Figure 1.**
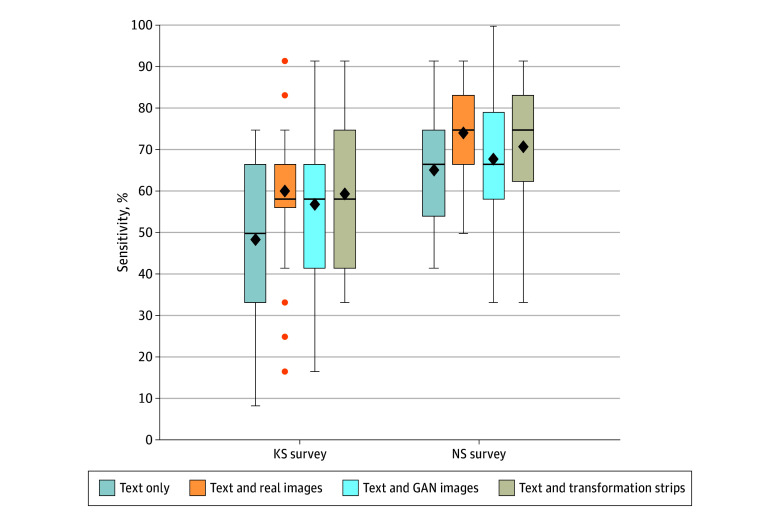
Participant Accuracy Classifying Kabuki Syndrome (KS) and Noonan Syndrome (NS) Images After Educational Interventions Mean accuracy (diamonds) increased with all image interventions compared with text description alone. The greatest accuracy increase was observed with KS, for which all types of images (real, generative adversarial network [GAN], and transformation strips) yielded the same median value (horizontal bar inside the boxes, with the lower and upper ends of the boxes indicating the first and third quartiles) and averages nearly 10% higher than text description alone. The whiskers indicate values within 1.5 × the IQR from the upper or lower quartile (or the minimum and maximum if within 1.5 × the IQR of the quartiles). Data more extreme than the whiskers are plotted individually as outliers (circles).

Of 2515 individuals contacted, 106 and 102 pediatric residents completed the KS and NS surveys, respectively (mean [SD] postgraduate [PGY] years, 2.09 [0.86]) (eTable 2 in Supplement 2). We analyzed the KS and NS surveys separately. For each of these 2 conditions, we further partitioned the survey data into 2 subsets: condition specific (only KS or NS) and other condition images. This approach allowed us to measure how interventions affect sensitivity (true-positive rate) (Figure 1) and specificity (true-negative rate).^31^

For KS, averaging all completed questions, text-only accuracy was 48.5% (128 of 264); the sensitivity of text only was not significantly different from random guessing (OR, 0.94; 95% CI, 0.69-1.29; *P* = .71) (eTable 3 in Supplement 1). We therefore fit the model without the intercept term, effectively treating the OR of text only as the same as random guessing (eg, OR = 1). The image interventions are then compared against this random-guessing reference point in Table 1. Compared with random guessing, the real images (60.3% [188 of 312]; OR, 1.52; 95% CI, 1.15-2.00; *P* = .003) and 2 types of images created by generative AI (57.0% [212 of 372]; OR, 1.32; 95% CI, 1.04-1.69; *P* = .02 and 59.6% [193 of 324]; OR, 1.47; 95% CI, 1.12-1.94; *P* = .006) statistically increased the sensitivity (Table 1). A noninferiority test on specificity showed that, with respect to the real-image intervention, the GAN (OR, 0.87; 95% CI, 0.60-1.26; *P* = .47) and transformation strip (OR, 0.97; 95% CI, 0.66-1.44; *P* = .88) interventions were not associated with a statistically worse result (eTable 4 in Supplement 1).

**Table 1.** Comparison of Association Strength Between Image Interventions and Sensitivity With Respect to the Text-Only Intervention

	Text only	Text and real images	Text and GAN images	Text and transformation strips
**Kabuki syndrome**				
Mean accuracy, No./total No. (%)^a^	128/264 (48.5)	188/312 (60.3)	212/372 (57.0)	193/324 (59.6)
OR (95% CI)^b^	1 [Reference]^c^	1.52 (1.15-2.00)	1.32 (1.04-1.69)	1.47 (1.12-1.94)
*P* value	NA	.003	.02	.006
**Noonan syndrome**				
Mean accuracy, No./total No. (%)^a^	196/300 (65.3)	205/276 (74.3)	204/300 (68.0)	247/348 (71.0)
OR (95% CI)^b^	1.89 (1.48-2.40) [Reference]	1.53 (1.08-2.18)	1.13 (0.77-1.66)	1.30 (0.92-1.83)
*P* value	<.001	.02	.54	.14

Abbreviations: GAN, generative adversarial network; NA, not applicable; OR, odds ratio.

^a^ Mean accuracy (true-positive rate) was computed by averaging the accuracy of each participant every time a Kabuki syndrome (and likewise Noonan syndrome) image was shown.

^b^ The OR for the text-only intervention is compared against random guessing, and the ORs for the 3 image interventions are compared against the text-only intervention.

^c^ The text-only intervention regression coefficient was not significantly different from 0 (eTable 3 in Supplement 1). Hence, we fit the model without the intercept term, effectively comparing the other image interventions against the OR of random guessing for binary output (eg, OR = 1).

For NS, the sensitivity analysis and noninferiority test demonstrated that only the real-image intervention statistically improved the sensitivity with respect to the text-only intervention (Table 1) and GAN and transformation strip intervention did not statistically lower the sensitivity with respect to the real-image intervention. The sensitivity for the NS text-only description was 65.3% (196 of 300). Compared with text only, the sensitivity of the real images was 74.3% (205 of 276; OR, 1.53; 95% CI, 1.08-2.18; *P* = .02), and the sensitivity of the 2 types of images created by generative AI was 68.0% (204 of 300; OR, 1.13; 95% CI, 0.77-1.66; *P* = .54) and 71.0% (247 of 328; OR, 1.30; 95% CI, 0.92-1.83; *P* = .14).

We note that completion rate is important in the context of noninferiority.^33^ Among the interventions, completion rates were similar. For KS, 22 of 25 (88%) completed the text-only portion, 26 of 27 (96.3%) completed the text and real images portion, 31 of 34 (91.2%) completed the text and GAN images portions, and 27 of 29 (93.1%) completed the text and transformation strips portions. For NS, 25 of 27 (92.6%) completed the text-only portion, 23 of 23 (100%) completed the text and real images portion, 25 of 27 (92.6%) completed the text and GAN images portions, and 29 of 29 (100%) completed the text and transformation strips portions (eTable 5 in Supplement 1).

For specificity, for both the KS and NS surveys, we did not observe any interventions to be significantly different from the text-only intervention (eTable 6 in Supplement 1). Therefore, we did not perform a noninferiority test as in the sensitivity analysis. Our result implies that by showing the participants example images (whether real or fake), we did not alter their log odd of ability to identify conditions besides KS and NS, respectively (ie, we observed no increase in the false-positive rates for identifying KS and NS images).

### Perceived Usefulness of Educational Intervention

Participants were also asked to rate the usefulness of the interventions. Approximately 60% of text-only description recipients found this useful for KS and NS. The reported usefulness of text increased when coupled with real images (Table 2). For example, the text descriptions were considered useful by more participants when coupled with real KS (20 [76.9%]) or NS (18 [78.3%]) images. A total of 25 of 26 KS survey participants (96.2%) found the real images useful, whereas 20 of 31 participants (64.5%) to 20 of 27 participants (74.1%) found the fake images useful. Similar findings were observed with NS participants.

**Table 2.** Usefulness of Text Descriptions and Image Intervention in Perceived Kabuki Syndrome and Noonan Syndrome Survey Performance

Group	Kabuki syndrome, No. (%)				Noonan syndrome, No. (%)			
	Text only (n = 22)	Text and real images (n = 26)	Text and GAN images (n = 31)	Text and transformation strips (n = 27)	Text only (n = 25)	Text and real images (n = 23)	Text and GAN images (n = 24)^a^	Text and transformation strips (n = 29)
Participants who found text description useful	13 (59.1)	20 (76.9)	18 (58.1)	18 (66.7)	15 (60.0)	18 (78.3)	15 (62.5)	17 (58.6)
Participants who found image intervention useful	NA	25 (96.2)	20 (64.5)	20 (74.1)	NA	21 (91.3)	19 (79.2)	22 (75.9)
Text-only intervention participants who would have found images to be useful	18 (81.8)	NA	NA	NA	25 (100)	NA	NA	NA

Abbreviations: GAN, generative adversarial network; NA, not applicable.

^a^ One participant did not complete questions about usefulness.

### Participant Confidence Levels

Although participants reported a range of confidence in identifying the genetic conditions, they were less confident for KS, with 94 of 106 (88.7%) reporting being not confident vs 52 of 102 (51.0%) for NS (eTable 2 in Supplement 2). The number of participants who reporting being confident or somewhat confident for NS was similar from PGY-1 to PGY-3 participants (range, 13 of 29 [44.8%] to 14 of 29 [48.3%]). All PGY-4 participants in the NS survey (n = 4) were confident or somewhat confident.

When a participant rated their answer as highly confident or confident, the mean accuracy for these questions was higher than when less confident (Table 3). For KS, for all interventions, no or low confidence (the reference) was not statistically significant, suggesting that when lacking confidence, results are not different from random guessing. For text only, even when participants felt confident, answers were not better than when they felt not or low confidence (OR, 1.45; 95% CI, 0.72-2.94; *P* = .30). For image-based interventions, there was a positive association between confidence level and accuracy (ie, when the participant felt confident, their answers were more likely to be correct).

**Table 3.** Association Between Confidence Level and Sensitivity for the 12 Condition-Specific Images in Each Survey

	Text only		Real images		GAN images		Transformation strips	
	Not or somewhat confident	Confident or highly confident	Not or somewhat confident	Confident or highly confident	Not or somewhat confident	Confident or highly confident	Not or somewhat confident	Confident or highly confident
**Kabuki syndrome**								
Mean accuracy, No./total No. (%)^a^	102/216 (47.2)	26/46 (56.5)	120/229 (52.4)	67/82 (81.7)	135/275 (49.1)	75/95 (78.9)	136/244 (55.7)	59/79 (74.7)
OR (95% CI)	0.89 (0.62-1.29) [Reference]	1.45 (0.72-2.94)	1.12 (0.85-1.48) [Reference]	3.99 (1.92-8.25)	0.97 (0.75-1.26) [Reference]	3.86 (2.05-7.28)	1.22 (0.89-1.68) [Reference]	2.31 (1.18-4.50)
*P* value	.55	.30	.42	<.001	.83	<.001	.22	.01
**Noonan syndrome**								
Mean accuracy, No./total No. (%)^a^	133/217 (61.3)	63/83 (75.9)	107/158 (67.7)	98/116 (84.5)	121/197 (61.4)	83/103 (80.6)	154/244 (63.1)	93/104 (89.4)
OR (95% CI)	1.58 (1.19-2.11) [Reference]	1.99 (1.00-3.94)	1.98 (1.51-2.60) [Reference]	2.75 (1.57-4.82)	1.59 (1.20-2.11) [Reference]	2.61 (1.47-4.63)	1.71 (1.35-2.17) [Reference]	4.94 (1.93-12.64)
*P* value	.002	.049	<.001	<.001	.001	.001	<.001	<.001

Abbreviations: GAN, generative adversarial network; OR, odds ratio.

^a^ The mean accuracy is given in correct over total number of responses for all syndrome-specific questions (Kabuki syndrome or Noonan syndrome) for all participants in each educational intervention group. One participant in each group did not answer 1 or more confidence questions.

For all NS interventions, even when not confident, participants guessed the correct answer more frequently than random chance (eg, log odd is >1) (Table 3). However, there was a positive association between confidence and accuracy. Text-only intervention showed minimal sensitivity improvement when confident (OR, 1.99; 95% CI, 1.00-3.94; *P* = .049) (Table 3). For real image, GAN, and transformation interventions, the sensitivity significantly improved with respect to confidence level.

### Perceptions About Facial Features

**Figure 2.**
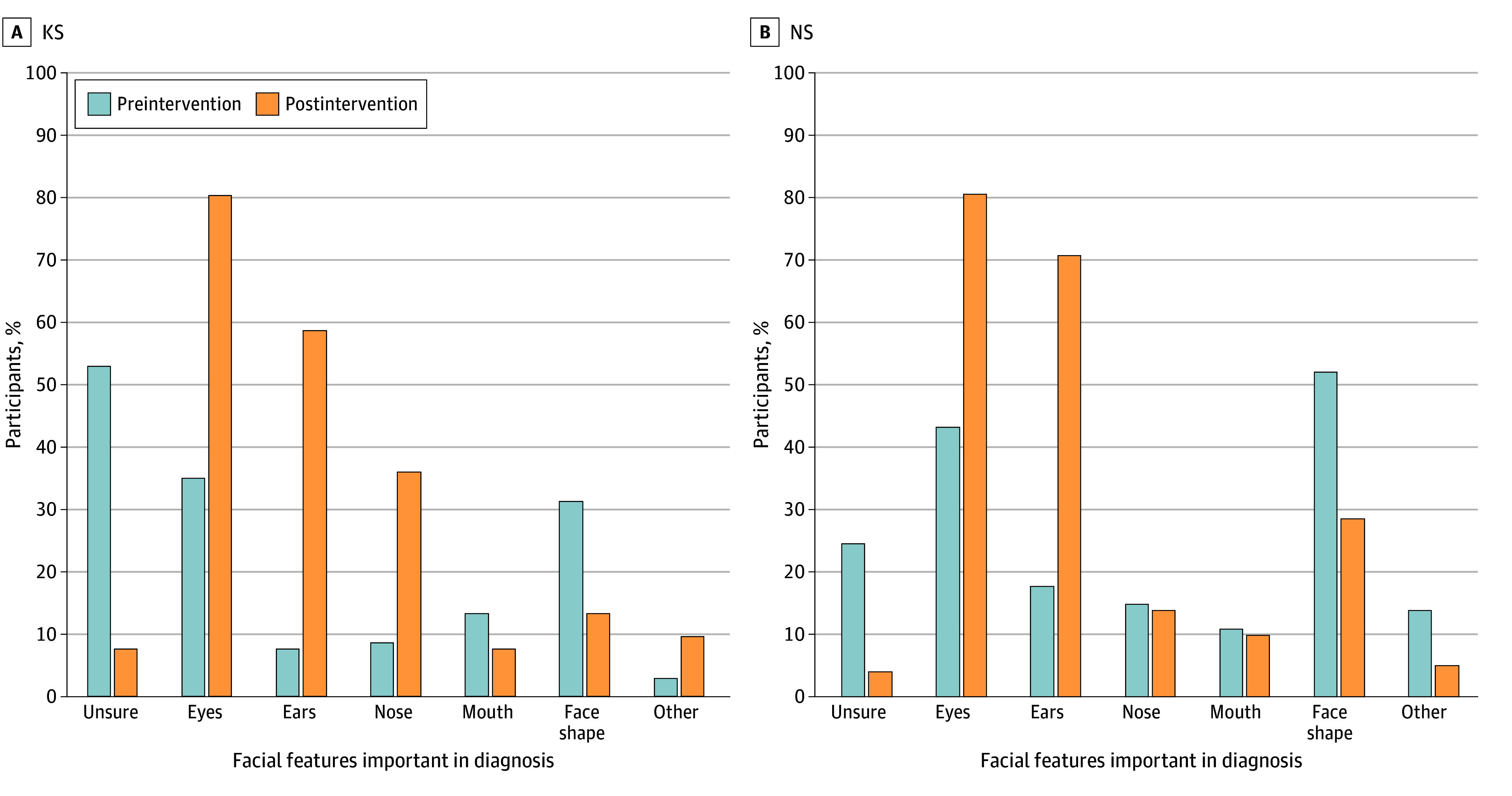
Self-Reported Facial Features Important in Diagnosis Before and After Intervention All interventions increased awareness of important facial features in both Kabuki syndrome (KS) and Noonan syndrome (NS), with a decrease in unsure response and an increase in typical dysmorphic features (eyes, ears, and nose for KS and eyes and ears for NS).

Participants were asked before educational interventions and at the end of surveys about which facial features were important (Figure 2). A total of 56 of 106 KS survey participants (52.8%) reported being unsure about important diagnostic facial features, whereas 25 of 102 (24.5%) NS survey participants were unsure. At the survey conclusion, the number of participants who reported being unsure decreased to 8 of 106 (7.5%) for KS (*P* < .001) and 4 of 102 (3.9%) for NS (*P* < .001). Regardless of intervention, the number of participants who selected the appropriate facial features increased. For KS, the greatest changes were observed in the correct selection of eyes, ears, and nose, which was overall greater in participants receiving image interventions (eFigure 3 in Supplement 1). The greatest changes between presurvey and postsurvey for NS was an increase in correctly reporting eyes and ears as important features and a decrease in reporting the mouth as important (eFigure 4 in Supplement 1).

### Impact of Age, Ancestry, and Gender on Syndromic Features

To explore the perceived impact of age, ancestry, and gender, we asked participants to report how much each factor influences the facial features of each syndrome, once before the educational intervention and once after classifying all images (ie, we did not ask about individual images).

Before the interventions, 76 of 105 (72.4%) to 83 of 106 (78.3%) of KS and 34 of 102 (33.3%) to 45 of 102 (44.1%) of NS survey respondents reported being unsure of these factors’ influences. After the survey, all participants reported an opinion about the influence of these factors (eTable 7 in Supplement 2). For both surveys, those receiving the text-only intervention reported gender as having more influence on facial features (15 of 22 [68.3%] and 22 of 25 [88%]) than the mean of those receiving text plus any image (40 of 83 [48.2%] and 49 of 76 [64.5%]) for KS and NS, respectively).

## Discussion

There is excitement around AI developments, but this excitement is tempered by concerns, including that careful testing needs to be performed to ensure that the benefits outweigh risks. This study tested 2 key measures: participants’ opinions and self-reported confidence as well as changes in accuracy. Accuracy for NS improved modestly with images, whereas for KS, accuracy improved more greatly when images were included (Figure 1). The more modest increases in accuracy with the addition of images observed in NS could be due to greater participant familiarity with NS, which is supported by the higher text-only accuracy and confidence levels for NS vs KS.

Most participants rated all image types as useful, with real images rated most useful. The study found a discordance in perceived usefulness and performance, which may reflect intrinsic differences between real and GAN images but may also involve a bias against fake images because participants were aware of whether images were real or generated. Future work could explore this area further.

After exposure to images of syndromic individuals, participants were more likely to express an opinion about important facial features (Table 2) and the influences of age, gender, and ancestry on syndromic facial features (eTable 7 in Supplement 2). This finding is encouraging because exposure could lead to more informed perspectives. We caution that further study with a larger sample size would be required to better understand these trends.

In our study, the use of images to supplement text descriptions resulted in similar or better accuracy vs text-only interventions, but real images performed better than generated images for both KS and NS. Interestingly, for KS, generated images were noninferior with respect to real images (eTable 4 in Supplement 1). Our results imply that, if participants are unfamiliar with the genetic condition and if there are insufficient real images available, then the use of AI-generated images may be a helpful adjunct. Advantages of generative AI include that many and diverse images can be made quickly. Further study is needed to ensure that tools work equitably.^34,35^ Generative AI can also help address privacy and data-sharing issues. Finally, our (and other) results also show that realistic images can be generated with relatively small data sets, such as may be available for rare diseases.^6,24,25^

We are aware that bias and inaccuracies are important problems in AI, including related to generative AI, and taking steps to mitigate these issues are important to consider. In addition to attempting to train and test the models using a variety of images, we took steps to manually review generative output to help provide manual checks regarding the accuracy of output. A team of at least 2 genetic clinicians selected generated images that most accurately represented KS and NS. In future studies, we plan to further validate potential biases and inaccuracies when using AI without such human intervention.

### Limitations

Our study has limitations. We examined only 2 conditions and had a small number of participants. We asked participants to classify images only after intervention, with no preintervention assessment. Additionally, we only tested online surveys vs other techniques. Further study to include more conditions, participants, learning modalities, and different types of data could provide a broader sense of these approaches.

## Conclusions

In this study, AI-generated images were associated with improvements in pediatric residents’ ability to recognize KS and NS. The findings of this comparative effectiveness analysis do not imply that generative AI could replace traditional teaching methods but describe an opportunity for AI-human collaboration to enhance genetics education. In this context, AI might best be viewed as another means to address different learning styles and provide additional content in new ways. These approaches can be useful in residency programs with little contact with medical geneticists.

## Notes

Supplement 1. **eFigure 1.** Generation of Unaffected and Syndromic Faces **eMethods.** Supplemental Methods **eFigure 2.** Schema of Kabuki Syndrome Surveys **eTable 3.** Logistic Regression for Clustered Data Fit With and Without the Text-Only Intervention as the Intercept Term **eFigure 3.** Pre- to Post-Intervention Change in Important Diagnostic Facial Features of Kabuki Syndrome **eFigure 4.** Pre- to Post-Intervention Change in Important Diagnostic Facial Features of Noonan Syndrome **eTable 4.** Non-inferiority Analysis for KS and NS Image Interventions **eTable 5.** Survey Completion Rates **eTable 6.** Comparing Association Strength Between Image-Interventions and Specificity With Respect to the Text-Only Intervention
eReferences

Supplement 2. **eTable 1.** Data Sources Used for Model Training and Fine-Tuning **eTable 2.** Demographic Information About Participants **eTable 7.** Self-Reported Influence of Age, Gender, and Ancestry on Syndromic Facial Characteristics Prior to and After Interventions

Supplement 3.
Data Sharing Statement
