# Supplementary material for: Recognition of Genetic Conditions After Learning With Images Created Using Generative Artificial Intelligence
Source: JAMA Netw Open. 2024 Mar 15;7(3):e242609. doi: 10.1001/jamanetworkopen.2024.2609 (PMC10943405; doi:10.1001/jamanetworkopen.2024.2609)
Supplement: Supplement 1. — eFigure 1. Generation of Unaffected and Syndromic Faces eMethods. Supplemental Methods eFigure 2. Schema of Kabuki Syndrome Surveys eTable 3. Logistic Regression for Clustered Data Fit With and Without the Text-Only Intervention as the Intercept Term eFigure 3. Pre- to Post-Intervention Change in Important Diagnostic Facial Features of Kabuki Syndrome eFigure 4. Pre- to Post-Intervention Change in Important Diagnostic Facial Features of Noonan Syndrome eTable 4. Non-inferiority Analysis for KS and NS Image Interventions eTable 5. Survey Completion Rates eTable 6. Comparing Association Strength Between Image-Interventions and Specificity With Respect to the Text-Only Intervention eReferences [file jamanetwopen-e242609-s001.pdf]

Supplementary Online Content

Waikel RL, Othman AA, Patel T, et al. Recognition of genetic conditions after learning with images created using generative artificial intelligence. *JAMA Netw Open*. 2024;7(3):e242609. doi:10.1001/jamanetworkopen.2024.2609

eFigure 1. Generation of Unaffected and Syndromic Faces

eMethods. Supplemental Methods

eFigure 2. Schema of Kabuki Syndrome Surveys

eTable 3. Logistic Regression for Clustered Data Fit With and Without the Text-Only Intervention as the Intercept Term

eFigure 3. Pre- to Post-Intervention Change in Important Diagnostic Facial Features of Kabuki Syndrome

eFigure 4. Pre- to Post-Intervention Change in Important Diagnostic Facial Features of Noonan Syndrome

eTable 4. Non-inferiority Analysis for KS and NS Image Interventions

eTable 5. Survey Completion Rates

eTable 6. Average Survey Accuracies for the 8 Other Condition Images Stratified Survey Type

eReferences

This supplementary material has been provided by the authors to give readers additional information about their work.

© 2024 Waikel RL et al. *JAMA Network Open.*

eFigure 1. **Generation of unaffected and syndromic faces.** First, real images were aligned so that all the faces are roughly in the same size and orientation. StyleGAN2-ADA was finetuned with disease, age, and gender as labels; this provides us more control at generating images within a specific category. The right side shows the transformations of fake unaffected images into Kabuki syndrome. ADA: adaptive discriminator augmentation.

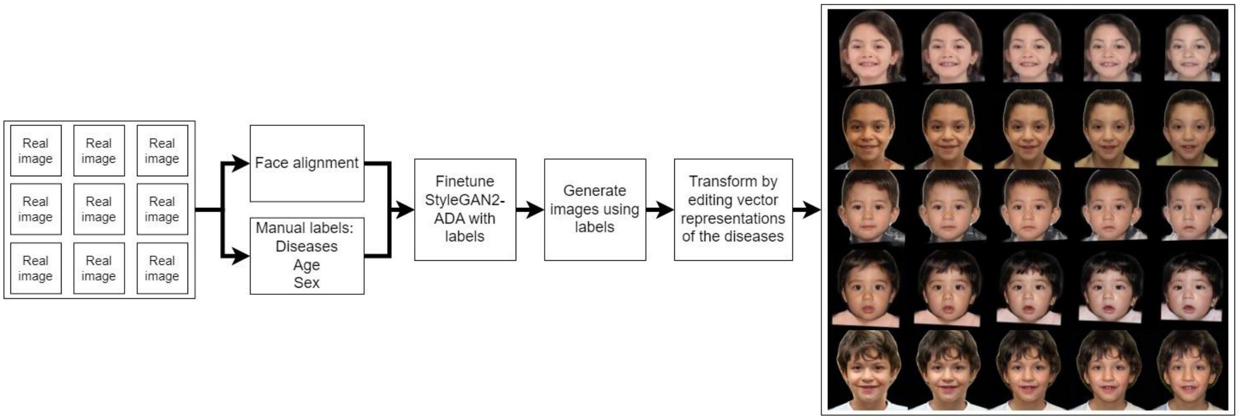

© 2024 Waikel RL et al. *JAMA Network Open.*

eMethods. Supplemental Methods

Detailed methods for generative AI images

Our image generator is based on Nvidia StyleGAN2-ADA. StyleGAN2-ADA creates a fake random image by converting a random vector into this image;[1] a label vector (default size 512x1), if available, can also be used with this random vector, providing the user more control for generating images with certain criteria (e.g., face images of only young individuals). The default Nvidia StyleGAN2-ADA trained on FFHQ dataset did not use labels. We followed the same approach as in our previous work,[2,3] and finetuned the default StyleGAN2-ADA on our own labeled datasets. Our label vector of size 512 consists of three subparts: a vector of size 256 indicating the disease label, a vector of size 128 indicating the age group, and a vector of size 128 indicating the gender. Hence, the additional parameters to be trained are the disease embedding 256 x 11 (we used 10 genetic conditions and one set of unaffected images training (see: https://github.com/datduong/stylegan3-syndromic-faces)), the age embedding 128 x 5 for five age groups (under 2 years old, 2-9 years old, 10-19 years old, 20-34 years old, and >35 years old), and gender embedding 128 x 2 for female and male. Due to low sample sizes, we could only train a small number of embeddings. Hence, the age and gender embedding are shared across all images; that is, these embeddings are not specific to a particular genetic condition.

Although we evaluated the GAN application on just KS and NS, we used images of the other conditions during fine-tuning (eTable 1). Since people with some of these conditions can have similar facial features, using images of people with additional conditions allows us to generate more specific images of people with the genetic conditions of interest.

Because our dataset is small, we fine-tuned Nvidia StyleGAN2-ADA of images size 256 x 256 instead of the higher resolution 1024 x 1024. Following Nvidia image pre-processing, we aligned all the faces so that all images have roughly similar head sizes/orientations and eye positions. We further removed the background by setting these pixel values to zero; this approach helps remove some potential GAN artifacts such as artifactual strands of hair or backgrounds. Since we have different numbers of images of each condition, during fine-tuning we applied sample weighting so that images from each disease have roughly uniform weights (see: https://github.com/datduong/stylegan3-syndromic-faces).

We fine-tuned StyleGAN2-ADA on our images together with their corresponding labels (eFigure 1). These labels provide more control to generate fake images of a person with a particular genetic condition, age, and gender, and, for the "transformation strips" (see below), allow us to alter these fake images to resemble unaffected individuals by manipulating the disease embeddings. We generated single images as well as what we called "transformation strips", which depict an individual changing from unaffected to affected. This was inspired by previous work we had done using GANs to depict disease progression, such as cutaneous findings in neurofibromatosis type 1.[2] In preliminary testing, medical genetics residents reported transformation strips helped them recognize genetic conditions.

After fine-tuning, we selected a random vector and the label embeddings to generate a random image of a person representing a particular genetic condition, age group, and gender. To be more specific, such as to generate an image of young female individual with KS (and likewise NS), we would provide StyleGAN2-ADA with a random vector and the label embeddings of KS, young child, and female. To make this generated individual with KS look like a person without this condition (for the transformation strips

© 2024 Waikel RL et al. *JAMA Network Open.*

described above), we interpolated the embeddings of KS and unaffected, while keeping the corresponding random vector, the age, and gender embedding unchanged.

Preliminary testing with medical genetics residents

During a seminar, we asked ~20 clinical and laboratorian trainees enrolled in formal genetics training programs about the usefulness of these strips; all endorsed that they could be helpful. We next piloted three surveys, each with a different trainee. For classification of 14 real images, 14 GAN images, and 14 transformation strips, accuracy before and after viewing the images was: NS (real images): 85.7%, then 78.6%; NS (GAN images): 57.1%, then 85.7%; NS (transformation strips): 42.9%, then 85.7%; KS (real images): 85.7%, then 85.7%; KS (GAN images): 78.6%, then 85.7%; KS (transformation strips): 42.9%, then 57.1%.

Detailed statistical analyses

Logistic regression for clustered data was applied since each image was seen by multiple different participants. Following the notations in Miglioretti et al.[4], we have the following model:

$\mathrm{logit}\,\pi^M(Y_{ij}) = \beta^M_{text} + X_{ij,real}\beta^M_{real} + X_{ij,GAN}\beta^M_{GAN} + X_{ij,trans}\beta^M_{trans}$ (Equation 1)

$X_{ij,real}$, $X_{ij,trans}$, and $X_{ij,trans}$ are binary dummy variables indicating to which intervention the participant i was exposed prior to analyzing the image j.

$logit\,\pi^M(Y_{ij})$ denotes the logit transformation of the binary output indicating if a participant i correctly identifies the image j. π^M is the metric of interest (discussed below).

We analyze the KS and NS surveys separately. For each of these two conditions, we further partition the survey data into two subsets. For example, when analyzing KS surveys, we partition the questions into a subset containing only KS images, and then another subset containing only the other conditions. This allows us to measure how the inventions affect the sensitivity $\pi^M(Y_{ij}) = P(Y_{ij} = 1 | D_{ij} = 1, X_{ij})$ and specificity $\pi^M(Y_{ij}) = P(Y_{ij} = 0 | D_{ij} = 0, X_{ij})$, where D_{ij} indicates where or not the image j is indeed a patient with KS syndrome.

The intercept β^M_{text} corresponds to the marginal (or population-averaged) effect of the text-only intervention. The regression coefficients β^M_{real}, β^M_{GAN}, and β^M_{trans} estimate how the log odd changes with respect to the text-only intervention. These parameters were estimated using generalized estimation equations (GEE) via the R library geepack.[5] Table s1 and s2 show the sensitivity outcome of KS with and without the text-only intervention as the intercept term. Table s4 shows the sensitivity outcome of NS. Tables s3 and s5 show the noninferiority for each survey sensitivity analysis. Table s6 shows the specificity outcomes of both KS and NS.

Suppose at least one of β^M_{real}, β^M_{GAN} and β^M_{trans} is statistically non-zero, then we perform noninferiority test between GAN and transformation intervention against the real image intervention. Logically, we are interested in noninferiority test only when one of the alternative interventions statistically differs from the text-only intervention.[6]

For noninferiority test, we remove the text-only surveys, and analyze only the responses for the other three interventions. Similar to Equation 1, we fit the model:

$logit\,\pi^M(Y_{ij}) = \alpha^M_{real} + X_{ij,GAN}\alpha^M_{GAN} + X_{ij,trans}\alpha^M_{trans}$ (Equation 2)

© 2024 Waikel RL et al. *JAMA Network Open.*

Here, α_{real}^{M} is the intercept, measuring the marginal log odd of success in the real image intervention. α_{GAN}^{M}, and α_{trans}^{M} estimate how the other two interventions change the log odd with respect to the real image intervention. We again apply R library geepack to approximate the regression coefficients (Tables s2 and s4).

We applied the same regression technique as above to analyze the how confidence level correlates with accuracy within each intervention. Separately in each intervention, we test whether there is a positive association between confidence level and accuracy (Table y2 and y3).

**Sensitivity analysis KS survey**	Estimate	Std.err	Wald	Pr(>|W|)	CI 2.5%	CI 97.5%
β_{text}^{M}	-0.0606	0.1614	0.14	0.707	-0.377	0.256
β_{real}^{M}	0.4768	0.2142	4.96	0.026	0.057	0.897
β_{GAN}^{M}	0.342	0.2036	2.82	0.093	-0.0571	0.741
β_{trans}^{M}	0.4481	0.2145	4.37	0.037	0.0278	0.868

Table s1. Sensitivity analysis in KS survey, Equation 1 was fitted with all the regression coefficients, and the intercept β_{text}^{M} is found to be statistically insignificant.

**Sensitivity analysis KS survey**	Estimate	Std.err	Wald	Pr(>|W|)	CI 2.5%	CI 97.5%
β_{real}^{M}	0.4162	0.1408	8.741	0.00311	0.14027	0.692
β_{GAN}^{M}	0.2814	0.1242	5.136	0.02343	0.03804	0.5248
β_{trans}^{M}	0.3875	0.1412	7.531	0.00606	0.11074	0.6642

Table s2. Sensitivity analysis in KS survey. Equation 1 was fitted to the survey subset containing only questions showing other syndromes in the KS surveys. The intercept β_{text}^{M} was removed because it was not found to be statistically significant. Real, GAN, and transformation strip interventions (β_{real}^{M}, β_{GAN}^{M}, and β_{trans}^{M}) statistically improve the odd of recalling KS images, as compared to random guessing.

**Noninferiority test on sensitivity KS survey**	Estimate	Std.err	Wald	Pr(>|W|)	CI 2.5%	CI 97.5%
α_{real}^{M}	0.4162	0.1408	8.74	0.0031	0.14	0.692
α_{GAN}^{M}	-0.1347	0.1877	0.52	0.4728	-0.503	0.233
α_{trans}^{M}	-0.0287	0.1994	0.02	0.8857	-0.419	0.362

© 2024 Waikel RL et al. *JAMA Network Open.*

Table s3. Noninferiority test on sensitivity for KS. Equation 2 was fitted to the survey subset containing only questions showing KS patients, and using only responses in the real image, GAN image, and transformation strip intervention. α^{M}_{GAN} and α^{M}_{trans} have nonsignificant p-values, indicating that, with respect to the real image intervention, GAN and transformation intervention do not statistically change the odd of recalling KS images.

**Sensitivity analysis NS survey**	Estimate	Std.err	Wald	Pr(>|W|)	CI 2.5%	CI 97.5%
β^{M}_{text}	0.634	0.124	25.98	3.40E-07	0.3901	0.877
β^{M}_{real}	0.427	0.181	5.57	0.018	0.0724	0.781
β^{M}_{GAN}	0.12	0.197	0.37	0.542	-0.2659	0.506
β^{M}_{trans}	0.261	0.176	2.2	0.138	-0.0839	0.605

Table s4. Sensitivity analysis in NS survey. Equation 1 was fitted to the survey subset containing only questions showing other syndromes in the KS surveys. Text-only intervention has a statistically positive effect β^{M}_{text}, indicating that the participants on averages may already be more familiar with NS. Only the real image intervention
β^{M}_{real} statistically improves the odd of recalling NS images with respect to the text-only intervention.

**Noninferiority test on sensitivity NS survey**	Estimate	Std.err	Wald	Pr(>|W|)	CI 2.5%	CI 97.5%
α^{M}_{real}	1.06	0.131	65.38	6.70E-16	0.803	1.3174
α^{M}_{GAN}	-0.307	0.201	2.32	0.13	-0.701	0.0879
α^{M}_{trans}	-0.166	0.181	0.85	0.36	-0.52	0.1879

Table s5. Noninferiority test on sensitivity for NS. Equation 2 was fitted to the survey subset containing only questions showing NS patients, and using only responses in the real image, GAN image, and transformation strip intervention. With respect to the real image intervention, GAN and transformation intervention (α^{M}_{GAN} and α^{M}_{trans}) do not statistically change the odd of recalling NS images.

	Estimate	Std.err	Wald	Pr(>|W|)
KS Survey				
β^{M}_{text}	0.659	0.13	25.78	3.80E-07
β^{M}_{real}	0.22	0.201	1.2	0.27
β^{M}_{GAN}	-0.026	0.175	0.02	0.88
β^{M}_{trans}	-0.265	0.181	2.16	0.14
NS Survey				
	Estimate	Std.err	Wald	Pr(>|W|)
β^{M}_{text}	1.9924	0.2168	84.48	<2e-16
β^{M}_{real}	-0.0465	0.3334	0.02	0.89

© 2024 Waikel RL et al. *JAMA Network Open.*

β^M_{GAN}	-0.1771	0.3319	0.28	0.59
β^M_{trans}	-0.1231	0.3283	0.14	0.71

Table s6. Specificity analysis in the KS and NS surveys. For each disease type, Equation 1 was fitted to the survey subset containing only questions showing the other syndromes. None of the intervention is statistically significant, indicating that, with respect to the text-only intervention, none of the interventions statistically affect how the average participant recognizes the other syndromes.

**Kabuki syndrome**	Confidence level	Estimate	Std.err	Wald	Pr(>|W|)	2.5%	97.5%
Text-only	No/low	-0.111	0.185	0.36	0.55	-0.474	0.252
	Med/high	0.374	0.36	1.08	0.3	-0.332	1.079
Real images	No/low	0.114	0.141	0.65	0.4199	-0.163	0.39
	Med/high	1.383	0.372	13.8	0.0002	0.653	2.11
GAN images	No/low	-0.029	0.133	0.05	0.83	-0.29	0.232
	Med/high	1.351	0.323	17.44	3.00E-05	0.717	1.985
Transformation	No/low	0.197	0.163	1.47	0.225	-0.122	0.516
	Med/high	0.836	0.341	6.01	0.014	0.168	1.504

Table s7. Logistic regression measuring the confidence level versus accuracy score within each educational intervention for KS survey. GEE method was applied to handle clustered data. No to some confidence (no/low) is set as the intercept, and confident to highly confident (med/high) is set as the slope**.** In all 4 interventions, the intercept term is statistically insignificant, indicating that when confidence is low, the participant randomly guesses the syndrome (e.g., guessing between "KS" and "other condition"). In the text-only intervention, the regression coefficient of med/high level is statistically insignificant, hence, even when the participant is confident, their log odd is still not different from random guessing. This is not the case for real-image, GAN, and transformation intervention. The 3 image interventions have significant med/high confidence-level coefficients; hence, when average participant was confident, they selected the correct syndrome with log odd statistically better than random guessing.

**Noonan syndrome**	Confidence level	Estimate	Std.err	Wald	Pr(>|W|)	2.5%	97.5%
Text-only	No/low	0.46	0.147	9.77	0.0018	0.1713	0.748
	Med/high	0.688	0.349	3.88	0.0487	0.0038	1.372
Real images	No/low	0.684	0.139	24.3	8.10E-07	0.412	0.955
	Med/high	1.011	0.286	12.5	0.00041	0.45	1.572

© 2024 Waikel RL et al. *JAMA Network Open.*

GAN images	No/low	0.465	0.145	10.3	0.0013	0.181	0.749
	Med/high	0.958	0.293	10.7	0.0011	0.384	1.532
Transformation	No/low	0.537	0.12	19.9	8.00E-06	0.301	0.773
	Med/high	1.598	0.479	11.1	0.00086	0.658	2.537

Table s8. Logistic regression measuring the confidence level versus accuracy score within each educational intervention for NS survey. GEE method was applied to handle clustered data. In each intervention, confidence level is positively correlated with accuracy.

© 2024 Waikel RL et al. *JAMA Network Open.*

eFigure 2. Schema of Kabuki syndrome surveys. Each condition evaluated had 4 arms (4 different educational interventions): text describing facial characteristics (no images), text plus 5 real images, text plus 5 GAN single images, and text plus 5 transformation strips. After the educational intervention, participants were asked to classify 20 images (12 syndrome of interest and 8 other syndromes). The same schema was used for Noonan syndrome. GAN: generative adversarial network.

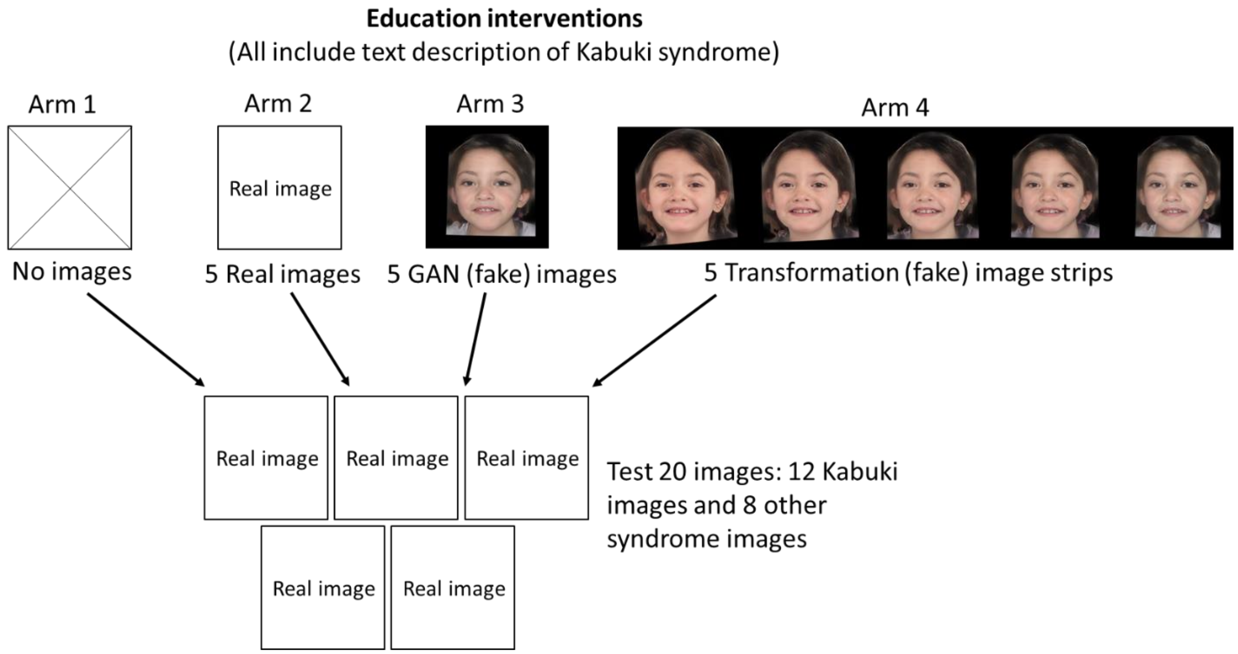

© 2024 Waikel RL et al. *JAMA Network Open.*

eTable 3. Logistic regression for clustered data fit with (left column) and without (right column) the text-only intervention as the intercept term. When fitting with the intercept term, the OR compares the text-only effect against random guessing. The OR of the other interventions were then compared against text-only. Without the intercept term, the reference OR is set as 1 to represent random guessing OR for binary output. The OR of the other interventions were then compared against random guessing.

	With intercept term	Without intercept term
**Text-only**		
Average Accuracy (%)	128/264 (48.5)	
OR (95% CI)	0.94 (0.69-1.29)<br>[Reference]	1<br>[Reference]
p-value	0.707	NA
**Real images**		
Average Accuracy (%)	188/312 (60.3)	
OR (95% CI)	1.61 (1.06-2.45)	1.52 (1.15-2.00)
p-value	0.026	0.003
**GAN images**		
Average Accuracy (%)	212/372 (57.0)	
OR (95% CI)	1.41 (0.95-2.10)	1.32 (1.04-1.69)
p-value	0.093	0.023
**Transformation strips**		
Average Accuracy (%)	193/324 (59.6)	
OR (95% CI)	1.57 (1.03-2.38)	1.47 (1.12-1.94)
p-value	0.037	0.006

© 2024 Waikel RL et al. *JAMA Network Open.*

eFigure 3. Pre- to post-intervention change in important diagnostic facial features of Kabuki syndrome. All interventions, including the text-only, involved a decrease in the “unsure” response and an increase in responses indicating the typical features associated with Kabuki syndrome (affecting the eyes, ears, and nose). GAN: generative adversarial network.

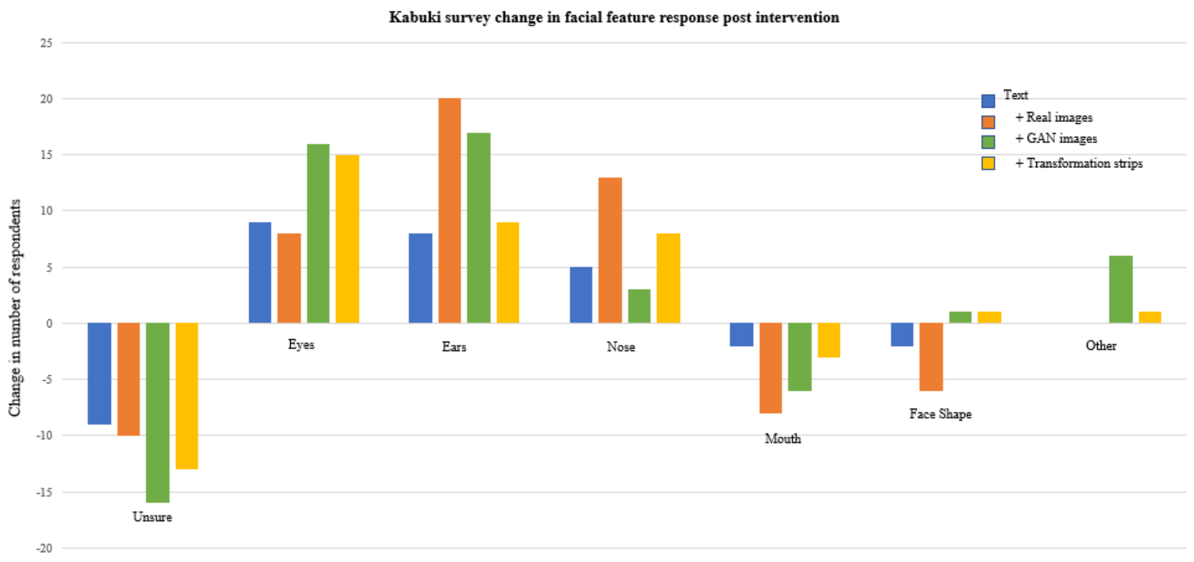

eFigure 4. Pre- to post-intervention change in important diagnostic facial features of Noonan syndrome. All interventions, including the text-only, involved a decrease in the “unsure” response and an increase in responses indicating the typical features associated with Noonan syndrome (affecting the eyes and ears). GAN: generative adversarial network.

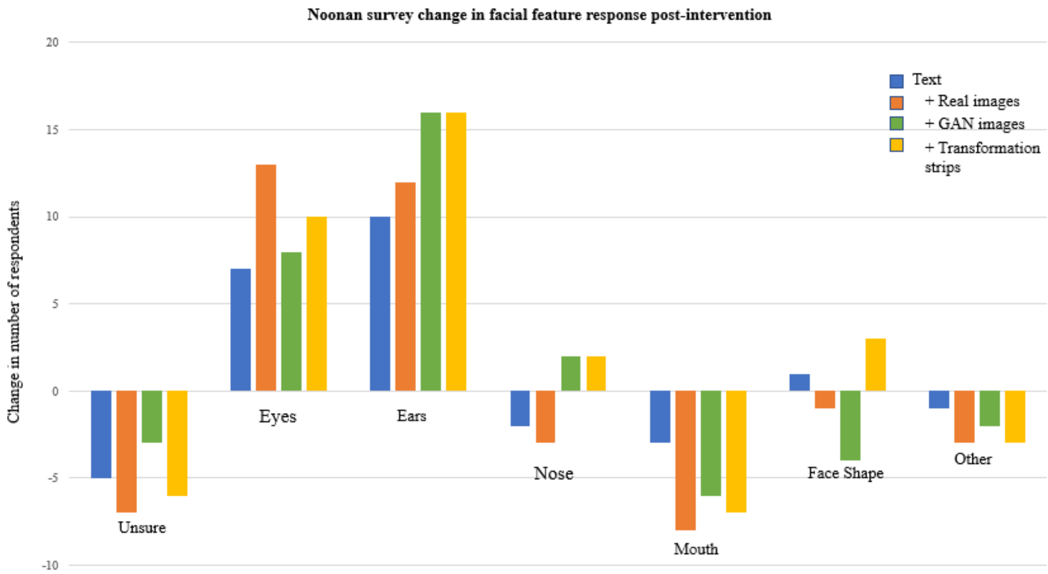

© 2024 Waikel RL et al. *JAMA Network Open.*

eTable 4. Non-inferiority analysis for KS and NS image interventions. OR were calculated for the real images compared to random chance (e.g. viewing real images are associated with a 1.52 times higher odds of accuracy than random guessing). Generative images were compared to real images for each condition. Although, generative images have a lower OR than real images, these differences were not statistically significant. OR: odds ratio; CI: confidence interval; GAN: generative adversarial network.

	Text + Real images	Text + GAN images	Text + Transformation Strips
**Kabuki syndrome**			
Average Accuracy (%)	188/312 (60.3)	212/372 (57.0)	193/324 (59.6)
OR (95% CI)	1.52 (1.15-2.00) [Reference]	0.87 (0.60-1.26)	0.97 (0.66-1.44)
p-value	0.0031	0.4728	0.8857
**Noonan syndrome**			
Average Accuracy (%)	205/276 (74.3)	204/300 (68.0)	247/348 (71.0)
OR (95% CI)	2.89 (2.23-3.73) [Reference]	0.74 (0.50-1.09)	0.85 (0.59-1.21)
p-value	6.70E-16	0.13	0.36

© 2024 Waikel RL et al. *JAMA Network Open.*

eTable 5. Survey completion rates. A survey was considered complete if a response was entered for all 20 test images. The classification portion of the survey was considered started if a response was entered for at least 1 test image. GAN: generative adversarial network.

	Text-only	Text + Real images	Text + GAN images	Text + Transformation Strips
**Kabuki syndrome**				
Participants who completed classification portion of survey/ started classification questions (%)	22/25 (88%)	26/27 (96.3)	31/34 (91.2)	27/29 (93.1)
**Noonan syndrome**				
Participants who completed classification portion of survey/ started classification questions (%)	25/27 (92.6)	23/23 (100)	25/27 (92.6)	29/29 (100)

© 2024 Waikel RL et al. *JAMA Network Open.*

eTable 6. Comparing association strength between image-interventions and specificity with respect to the text-only intervention. Average accuracy (true negative rate) was computed by averaging the accuracy of each participant every time an image of a syndrome other than the syndrome of interest was shown for both the KS and NS surveys. Odds ratios (OR) of text-only intervention are significantly higher than random guessing, and the other three interventions are not significantly different from the text-only intervention. CI: confidence interval; GAN: generative adversarial network.

	Text-only	Text + Real images	Text + GAN images	Text + Transformation Strips
**Kabuki syndrome**				
Average Accuracy (%)	116/176 (65.9)	147/208 (70.7)	162/248 (65.3)	129/216 (59.7)
OR (95% CI)	1.93 (1.50, 2.49) [Reference]	1.25 (0.84, 1.85)	0.97 (0.69, 1.37)	0.77 (0.54, 1.09)
p-value	3.80E-07	0.27	0.88	0.14
**Noonan syndrome**				
Average Accuracy (%)	176/200 (88)	161/184 (87.5)	172/200 (86.0)	201/232 (86.7)
OR (95% CI)	1.89 (1.48, 2.40) [Reference]	1.53 (1.08, 2.18)	1.13 (0.77, 1.66)	1.30 (0.92, 1.83)
p-value	<2e-16	0.89	0.59	0.71

© 2024 Waikel RL et al. *JAMA Network Open.*

eReferences

1. Jeong JJ, Tariq A, Adejumo T, Trivedi H, Gichoya JW, Banerjee I. Systematic Review of Generative Adversarial Networks (GANs) for Medical Image Classification and Segmentation. *J Digit Imaging.* 2022;35(2):137-152.
2. Duong D, Waikel RL, Hu P, Tekendo-Ngongang C, Solomon BD. Neural network classifiers for images of genetic conditions with cutaneous manifestations. *HGG Adv.* 2022;3(1):100053.
3. Duong D, Hu P, Tekendo-Ngongang C, et al. Neural Networks for Classification and Image Generation of Aging in Genetic Syndromes. *Front Genet.* 2022;13:864092.
4. Miglioretti DL, Haneuse SJ, Anderson ML. Statistical approaches for modeling radiologists' interpretive performance. *Acad Radiol.* 2009;16(2):227-238.
5. Højsgaard S, Halekoh U, Yan J. The R Package geepack for Generalized Estimating Equations. *Journal of Statistical Software.* 2005;15(2):1 - 11.
6. Cuzick J, Sasieni P. Interpreting the results of noninferiority trials—a review. *British Journal of Cancer.* 2022;127(10):1755-1759.

© 2024 Waikel RL et al. *JAMA Network Open.*
