# Supplementary material for: Recognition of Genetic Conditions After Learning With Images Created Using Generative Artificial Intelligence
Source: JAMA Netw Open. 2024 Mar 15;7(3):e242609. doi: 10.1001/jamanetworkopen.2024.2609 (PMC10943405; doi:10.1001/jamanetworkopen.2024.2609)
Supplement: Supplement 3. — Data Sharing Statement [file jamanetwopen-e242609-s003.pdf]

Data Sharing Statement

Waikel. Recognition of Genetic Conditions After Learning With Images Created Using Generative Artificial Intelligence. *JAMA Netw Open*. Published March 15, 2024.
doi:10.1001/jamanetworkopen.2024.2609

Data
Data available: Yes
Data types: Other (please specify)
Additional Information: Data and code have been made available through the paper, supplementary files, and via links on GitHub.
How to access data: https://github.com/datduong/stylegan3-syndromic-faces
When available: With publication

Supporting Documents
Document types: Statistical/analytic code
How to access documents: https://github.com/datduong/stylegan3-syndromic-faces
When available: With publication

Additional Information
Who can access the data: researchers whose proposed use of the data has been approved
Types of analyses: any reasonable research or educational purpose
Mechanisms of data availability: Code is available to anyone accessing the GitHub site. High resolution images will be provided with approval and investigator support.
